# Report on Otology

**Published:** 1875-01-15

**Authors:** Samuel J. Jones

**Affiliations:** Professor of Ophthalmology and Otology in the Chicago Medical College


### REPORT ON OTOLOGY.

BY SAMUEL J. JONES, M.D., PROFESSOR OF OPHTHALMOLOGY AND OTOLOGY IN THE CHICAGO MEDICAL COLLEGE.

*From Transactions of Illinois State Medical Society.*

IN presenting to the Society a Report for another year, on Otology, it has seemed desirable to devote a portion of it to the ear, in health, before proceeding to consider its diseases. The physician is often asked, by the laity, what care is necessary for the ear in health. In general terms, the proper answer is, none. The ear, like the other organs of the body, is so organized that, in health, it will take care of itself. When any one of its functions are so performed as to attract special attention, it is fair to assume that some abnormal condition exists, which renders it advisable that careful examination of the organ be made. The detection of the presence of an accumulation of cerumen in the external meatus, is a frequent source of chagrin to patients, who conclude at once that its existence argues a want of proper personal cleanliness. Such is not, however, necessarily the case. When the ear-wax is secreted in its normal condition, the ordinary movements of the jaws work it to the outer portion of the meatus, when the use of the towel in the bath will remove it. When its removal in this way fails to occur, examination will frequently reveal an unusually dry condition of the skin within the meatus. This dryness, in a large proportion of the cases, seems to be attributable to chronic non-suppurative inflammation of the ear. The wax secreted under these circumstances seems to be deficient in moisture, and, when it accumulates there will be found mixed with it, desquamated skin from the meatus—sometimes in quite large flakes; at other times, numerous small particles, and hairs that have been cast off with the epidermis. Having once begun thus to gather, the accumulation continues to increase; an additional portion of the watery part of the wax evaporates, leaving the mass harder. Gradually the meatus almost fills, and when illuminated there is a shining surface reflected, that has been mistaken, by those inexperienced in examining the ear, for the shining surface of the membrane of the drum of the ear. From this it may, however, readily be distinguished, by the fact that in the healthy membrane of the drum the reflected light shows itself in the form of a "triangular light spot," (which is absent when the membrane is opaque,) whereas the reflection from a mass of cerumen is an evenly-shining surface. To the experienced eye inspection will readily detect its existence, and various symptoms — usual consequences—of its presence, would suggest examination, such as impaired hearing—imaginary noises in the ear—sometimes dizziness, and unsteadiness of gait in walking, etc. When its existence has been discovered its removal may be most readily effected by syringing the meatus with some alkaline solution—alkalies being the most effectual solvent of the wax. Whatever fluids are injected into the meatus, their temperature should be very nearly that of the blood, to prevent either dizziness or inflammation resulting. Often this mass of wax is firmly adherent to the small hairs in the meatus which have not been cast off, and should an attempt be made to remove the mass by force, such violence may cause diffuse or circumscribed inflammation of the meatus. When this removal shall have been effected, inspection shows the unhealthy condition of the meatus. If the condition be one of dryness simply—such as occurs very frequently as an accompaniment of chronic catarrhal inflammation of the middle ear—lubricating the meatus with a solution of bi-borate of soda, with a small amount of glycerine and some carbolic acid added, is generally sufficient to save the patient much annoyance, and prevent a recurrence of the trouble until the removal of the primary cause affords entire relief.

The removal of foreign bodies from the meatus is often best effected by syringing—having first ascertained by inspection that such foreign body is present. If the foreign substance be an insect it can often be dislodged simply by filling the meatus with water, and thus cutting off its respiration; and in this way violence to the ear may be avoided. A word of caution regarding the use of an instrument designated an "aurilave," which is now being extensively vended, may not be amiss. Its construction is such that the piece of sponge that is attached to the end of the stick, when introduced into the meatus, forces the contents of the meatus before it, causing an impaction of the wax, epidermis, and loose hairs, in the inferior portion of the meatus, and often against the membrane of the drum of the ear.

The injudicious use of the nasal douche, is also an occasional source of injury to the healthy ear, producing acute inflammation of the middle ear, and perforation of the drum membrane. When being used the top of the column of fluid in the reservoir should be but a few inches above the nose.

Further observation seems to indicate very conclusively, that the ear, in health, possesses considerable power of accommodation, which fact may be taken advantage of to develop the power of hearing by systematic exercise, where any impairment exists.

Often the first departure from the condition of health, in the ears, which persons detect, is impairment of hearing, and of this they are accustomed to speak as a disease, instead of a consequence of disease. They often ask if their "deafness" is not hereditary, and state that certain other members of their family were sufferers from impaired hearing. Hereditary deafness, if possible, is certainly not of frequent occurrence. which fact, if more generally understood, would save much needless apprehension.

It is important that the distinction should be made between cases of impairment of hearing resulting from some defect in the conducting apparatus of the ear, and those in which the perceptive apparatus is at fault. Prognosis and treatment will both be materially influenced thereby.

In previous reports to the Society, allusion has been made to the gratifying diminution in the number of cases classed as nervous deafness. Recently attention has been more drawn to cases of total, or almost total deafness, following cerebro-spinal meningitis, and another form of affection of the labyrinth called Maniere's disease.

Professor Voltolini, of Breslau, has described what he calls "otitis labyrinthica," which occurs chiefly in children, and resembles in many respects the consequences which we have been accustomed to consider as cerebro-spinal meningitis.

It is a noticeable fact that the cases of deafness following cerebro-spinal meningitis seem to occur chiefly, if not exclusively, among quite young persons, and certain characteristics which he ascribes to otitis labyrinthica, should lead us to examine more closely our cases of supposed cerebro-spinal meningitis. In these cases the general practitioner has opened to him a large field of usefulness. The aural surgeon usually sees these cases only after all change has occurred, and loss of hearing has resulted, and an effort is being made for its restoration. More careful observation is needed in the beginning, and during the progress of the disease, and further, *post-mortem* examination of the labyrinth in fatal ones, to understand these mysterious cases. One of the embarrassing consequences of these diseases of the labyrinth, is the persistent tinnitus aurium. This ringing occurs under various circumstances and from various causes, so that treatment must, in each case, depend upon the cause which produces it. In some cases, where it was supposed to depend upon abnormal tension of the tensor tympani muscle, division of that muscle through the membrana tympani is said to have given relief.

The marked depression of spirits which occurs in persons who suffer from affections of the ear is a noticeable feature of these affections. It is most strikingly shown in those cases of persistent tinnitus, and in several recorded cases this is shown to have been the cause of suicide.

Your Committee would invite especial attention to acute inflammation of the mastoid cells, which is occasionally mistaken for erysipelas. The peculiar redness of the skin over the mastoid process, in these cases, is not unlike the appearance of erysipelas in its early stage. It is an error which occurs like that in acute inflammation of the lachrymal sac, and sometimes leads to disastrous consequences. It is sometimes mistaken for circumscribed, or even diffused, inflammation of the external meatus, which should not occur if the parts be at all carefully inspected. If the disease be recognized in its early stage the application of a few leeches over the mastoid process will generally prove sufficient to check its progress. If, however, the case be only seen at a later stage, when pus has formed, the sooner puncture is made and the pus evacuated the greater will be the safety to the patient. An anxious expression of countenance, in addition to the excessive pain accompanying this, characterizes it. Where discharge exists as a consequence of this inflammation, and escapes, either through a spontaneous or artificial opening, its removal should be hastened by thorough syringing with some astringent solution, as in the treatment of a discharge from the meatus. Regarding such discharge from the external meatus—so-called otorrhœa—there is a popular error that it is dangerous to check it lest metastasis to the brain occur. For fear that some such result might follow the checking of the discharge, it is allowed to continue for years, at the imminent risk to the life of the patient. That discharge is but a consequence of some pathological condition—usually the result of some acute or chronic inflammation—which should be discovered and removed, when the consequence will disappear with the removal of its cause. In every instance, without exception, where such discharge from the ear exists, an effort should at once be made to remove its cause. If there be perforation of the membrane of the drum of the ear accompanying it, on the disappearance of the discharge a decided tendency to closure of the opening in the membrane of the drum will manifest itself—contrary to the generally received opinion on that point. Where the edges of the opening are in apposition, or approximate it, the opening heals readily; if the opening be circular, or the edges wide apart, it may become necessary to cauterize them and start a granulating surface to fill the opening; but the tendency to repair is much greater than is generally supposed.

In diagnosis the contributions for the last year have probably been less than in some other departments of otology. A fact worthy of note is that, when the Eustachian tubes are inflated, either by the Valsalvian or Politzer's methods, or by the Eustachian catheter, and there is an improvement immediately in the patient's hearing, the difficulty lies, at least in part, in the Eustachian tubes. Where, however, the tubes are opened, the membrane of the drum is nearly normally clear, and no improvement in the hearing occurs on such inflation; the obstruction usually lies in some other portion of the conducting apparatus, or possibly in the perceptive apparatus, and the prognosis is more grave. For the purpose either of inflating the Eustachian tubes or of applying any medicament to the Eustachian tubes or middle ear, the pure silver catheter will be found the one most generally useful, as it can readily be bent at once to suit the variations of the nostrils of different patients, or of the two nostrils of the same patient. If properly adjusted it can be introduced without pain or danger to the patient, and it may safely be said that there is no substitute for it in the treatment of diseases of the ear.

Since the report of last year there has appeared a "Treatise on Diseases of the Ear," by Professor Roosa, of New York, which has no equal in the English language. It is a work useful alike to the aural surgeon and the general practitioner. A more limited work, entitled "Lectures on Diseases and Injuries of the Ear," by Mr. W. B. Dalby, of London, is also a valuable contribution, and embraces many of the more recently accepted ideas in aural surgery.

Numerous monographs on the various departments of otology have been written, which are valuable contributions to the literature of otology, and show the increasing interest now felt in this field, so much neglected in former years.

